# Association of physical function with connectivity in the sensorimotor and dorsal attention networks: why examining specific components of physical function matters

**DOI:** 10.1007/s11357-024-01251-8

**Published:** 2024-07-05

**Authors:** Madeline C. Boyd, Jonathan H. Burdette, Michael E. Miller, Robert G. Lyday, Christina E. Hugenschmidt, W. Jack Rejeski, Sean L. Simpson, Laura D. Baker, Chal E. Tomlinson, Stephen B. Kritchevsky, Paul J. Laurienti

**Affiliations:** 1 Department of Radiology, Wake Forest University School of Medicine, Medical Center Blvd, Winston-Salem, NC 27157 USA; 2 Division of Public Health Sciences, Wake Forest University School of Medicine, Winston-Salem, NC USA; 3 Sticht Center for Healthy Aging and Alzheimer’s Prevention, Department of Internal Medicine Section On Gerontology and Geriatric Medicine, Wake Forest University School of Medicine, Winston-Salem, NC USA; 4 Department of Health and Exercise Science, Wake Forest University, Winston-Salem, NC USA; 5 Department of Biostatistics and Data Science, Wake Forest University School of Medicine, Winston-Salem, NC USA; 6 Department of Biostatistics, University of North Carolina at Chapel Hill, Chapel Hill, NC USA; 7 Present Address: Janssen R&D of Johnson & Johnson, Raritan, NJ USA

**Keywords:** Brain networks, Magnetic resonance imaging, Community structure

## Abstract

Declining physical function with aging is associated with structural and functional brain network organization. Gaining a greater understanding of network associations may be useful for targeting interventions that are designed to slow or prevent such decline. Our previous work demonstrated that the Short Physical Performance Battery (eSPPB) score and body mass index (BMI) exhibited a statistical interaction in their associations with connectivity in the sensorimotor cortex (SMN) and the dorsal attention network (DAN). The current study examined if components of the eSPPB have unique associations with these brain networks. Functional magnetic resonance imaging was performed on 192 participants in the BNET study, a longitudinal and observational trial of community-dwelling adults aged 70 or older. Functional brain networks were generated for resting state and during a motor imagery task. Regression analyses were performed between eSPPB component scores (gait speed, complex gait speed, static balance, and lower extremity strength) and BMI with SMN and DAN connectivity. Gait speed, complex gait speed, and lower extremity strength significantly interacted with BMI in their association with SMN at rest. Gait speed and complex gait speed were interacted with BMI in the DAN at rest while complex gait speed, static balance, and lower extremity strength interacted with BMI in the DAN during motor imagery. Results demonstrate that different components of physical function, such as balance or gait speed and BMI, are associated with unique aspects of brain network organization. Gaining a greater mechanistic understanding of the associations between low physical function, body mass, and brain physiology may lead to the development of treatments that not only target specific physical function limitations but also specific brain networks.

**Supplementary Information:**

The online version contains supplementary material available at 10.1007/s11357-024-01251-8.

## Introduction

Age-associated loss of mobility compromises activities of daily living (ADLs) that are the foundation of older adults ability to remain independent in their communities [1]. Furthermore, loss of physical function is often a precursor to further mobility loss, pressure ulcers, falls, urinary incontinence, and malnutrition [2]. It is also well documented that obesity, assessed by body mass index (BMI), is a major risk factor for the loss of mobility [3]. A large and growing body of literature supports the importance of neural mechanisms in age-related mobility decline [4]. Most studies have examined these mechanisms in association with irreversible brain pathologies (e.g., white matter disease) [5, 6]. Less is known about the associations between physical function and brain networks in older adults and whether associations are moderated by BMI.

In the past few years, studies have begun to examine how functional brain networks relate to physical function in older adults. The sensorimotor network (SMN) is known to be involved in the processing of physical stimuli and coordinating motor responses. Low efficiency of local connectivity in the SMN in older adults is associated with poor gait stability [7]. Similarly, slow gait speed is associated with low levels of resting-state connectivity in the basal ganglia [8]. Brain networks associated with cognition may also be important in determining physical function. The dorsal attention network (DAN) is known to be involved in higher order motor coordination, such as balance and complex movement. Recently, it was demonstrated [9] that older adults with better performance on a manual visual-motor task have stronger connectivity within the SMN and DAN. Our previous work [10] showed that connectivity in the DAN was higher during a motor imagery task that engages visuospatial attention compared to resting state. SMN exhibited the opposite findings with connectivity decreased during the motor imagery task likely due to the desynchronization that occurs during motor imagery [11]. Another indication that cognition and cognition-related networks may be important determinants of physical function is that much of the emerging work examining the neural correlates of mobility decline has been in people with mild cognitive impairment (MCI), with poor physical function being associated with alterations in connectivity between the SMN and other brain regions [12–14].

Given the complexity of whole-brain networks, it is helpful to assess the connectivity of subnetworks within the context of the whole. Network modularity [15] is one of the most common methods to divide a network into communities such that the regions within each community are more interconnected with each other than with regions in other communities. Once networks have been divided into communities using modularity, it is possible to test hypotheses about how the spatial consistency of community structure in specific subnetworks varies between groups or by other measures of interest [16, 17].

Previously, we observed that the spatial pattern of the SMN and DAN communities was disrupted in people with poorer physical function, and this relationship was magnified as BMI increased [18]. The current study is an extension of that recent report of associations that both physical function and body mass index (BMI) have with functional brain network community structure within the SMN and DAN. Physical function was assessed by scores on the expanded Short Physical Performance Battery (eSPPB) [19], which is used to measure the ability of older adults to coordinate movement for ADLs. The four components of the eSPPB assess gait speed with a 4-m usual paced walk, static balance using multiple standing positions, lower extremity strength with a chair-stand test, and complex gait speed using a narrow walk test. Thus, it is a composite measure of gait speed, balance, and lower extremity strength.

The unique roles of the SMN and DAN in neural processes, as well as their relationships with physical function [20, 21], led to the following two hypotheses. Hypothesis 1: gait speed, lower extremity strength, and complex gait speed would be associated with the SMN as this circuit is known to generate motor commands and is essential for moving the lower extremities. Hypothesis 2: balance and complex gait speed would be associated with the DAN as this circuit controls spatial attention necessary for complex gait movements and balance. These associations would be most notable during a motor imagery task that involved spatial navigation through complex environments. In addition, associations were hypothesized between lower extremity strength and DAN connectivity as this it has been emphasized that perception and spatial attention are embodied processes [22] and strength in the lower extremities is highly relevant to the motor imagery task. It is predicted that all associations will be moderated by BMI such that higher subscale scores and lower BMI will be associated with stronger community structure.

Because the composite score of the eSPPB consists of four distinct subscales, we explore the moderating effects of BMI on each subscale to determine whether any observed effects are specific to particular domains of physical function. For example, studies have shown that BMI is inversely related to complex motor coordination; thus, domains of functions within the eSPPB such as complex gait speed and balance may have stronger interactions with BMI in their association with the community structure of the SMN and DAN. Analyses were run on brain networks generated using functional magnetic resonance imaging (fMRI) data of rest and motor imagery task conditions collected from participants in the Brain Networks and Mobility (B-NET) study (*n* = 192).

## Methods

### B-NET study design

B-NET (NCT0340427) was a longitudinal, observational trial of community-dwelling older adults (aged 70 and older) recruited from Forsyth County, NC, and surrounding areas. Recruitment took place via direct mailings, word of mouth, flyers, and a community newsletter distributed by the Sticht Center for Healthy Aging and Alzheimer’s Prevention at Wake Forest University School of Medicine. Each participant agreed to come in for two baseline visits and three follow-up visits over 30 months. The data used for this manuscript is from the baseline visits, which included brain MRIs, extensive health histories, and cognitive and physical function testing. The longitudinal data collection concluded in July of 2023.

### Participants

B-NET enrollment included 192 participants over the age of 70. Potential participants were excluded from the study on the basis of being a single or double amputee, having musculoskeletal implants that impeded functional testing (e.g., joint replacements), the inability/unwillingness to complete a brain MRI scan, and dependency on assistance for ambulation. Neurological/psychiatric exclusion criteria were as follows: clinical diagnosis of any disease affecting mobility (e.g., Parkinson’s disease), prior traumatic brain injury, history of brain tumor, recent history of seizures, diagnosis of any psychotic disorder, alcohol use disorder, or any evidence of impaired cognitive function as measured by the Montreal Cognitive Assessment (MoCA). A score of 20 or lower on the MoCA was considered exclusionary, and scores from 21 to 25 were reviewed by the study neuropsychologist to determine eligibility on an individual basis. Other exclusion criteria included hospitalization or surgery within the past 6 months, uncontrolled or serious chronic disease, uncorrected major hearing or vision problems, plans to relocate within 24 months, and active participation in a behavioral intervention trial. Our exclusions are in accordance with prior recommendations [23] to gain a clearer picture of age-related change without confounds associated with cognitive decline. For clarity on participants that were excluded from participation, a Strengthening the Reporting of Observational Studies in Epidemiology (STROBE) flow chart diagram [24] is included in the supplemental Methods (Figure S1). There were two participants missing the complex gait speed scores so analyses for this variable were limited to 190 participants with all other analyses included the full 192 participants. Extensive demographic characteristics were presented in a prior publication [18], and an abbreviated summary of characteristics relevant to the current study is presented in Table 1 of the “Results” section (Participant Characteristics). All participants gave written informed consent in this study as approved by the Wake Forest University School of Medicine Institutional Review Board (IRB, protocol #IRB00046460).

**Table 1:** Participant characteristics

*n*	192
Age (years)	76.4 (4.7)
Race	
Caucasian/White	173
African American/Black	18
American Indian/Alaskan Native	1
Asian	0
Ethnicity	
Hispanic/Latino	2
Sex	
Men	84
Women	108
BMI (kg/m^2^)	28.4 (5.6)
Physical function measures	
Balance score	0.72 (0.262)
Gait speed score	0.49 (0.100)
Complex gait speed score#	0.40 (0.193)
Lower extremity strength score	0.40 (0.119)
eSPPB score#	2.00 (0.523)

^#^Complex gait speed scores were not available for two participants. All statistical analyses for complex gait speed utilized 190 participants. The eSPPB score is based on 190 participants as it requires complex gait speed

### Baseline study measurements

At the first BNET baseline visit, all participants completed an extensive evaluation. Measures directly relevant to the current manuscript are as follows: self-reported age, sex, height, and weight. BMI was calculated using the participant’s height and weight measured using a wall-mounted stadiometer and a calibrated scale, respectively. The eSPPB was administered at the first visit, and MRI scans were collected during another baseline visit within approximately 1 month of the first visit; the eSPPB and MRI assessments are described below.

#### Expanded Short Physical Performance Battery (eSPPB)

The physical function of participants was assessed using the eSPPB test [19], which was adapted from the SPPB test developed by Guralnik et al. [25] to address effects that could limit the utility of the traditional SPPB in well-functioning populations. The original SPPB [25] assesses overall physical function by combining assessments of the following: (1) a 4-m timed usual pace walk; (2) a balance test involving side-by-side, semi-tandem, and full-tandem stances; and (3) repeated trials of standing up from sitting in a chair. The eSPPB expands the assessments to improve the sensitivity of the test in higher functioning older adults [19]. The four components of the eSPPB test allowed assessment of different dimensions of physical function. In the balance assessment (BAL), participants were asked to stand in a side-by-side posture for 10 s, and then hold the semi-tandem, tandem, and one-leg positions for 30 s each, reflecting individuals’ ability to coordinate movement and maintain balance. Two tests were used to measure gait: the participants’ gait speed during a 4-m walk (GS) and the participants’ “narrow walk” gait speed for a 4-m walk during which they were required to keep their steps between a set of parallel lines 20 cm apart (complex gait speed or CGS). The gait speed assessments are useful in determining individuals’ ability to ambulate effectively, and, in the narrow walk condition, to ambulate with more precision. To assess lower extremity strength (LES), participants were timed while standing up from a seated position five times without using their arms. This test evaluates the physical strength as well as how quickly participants can recover from a movement and how much repetition they can withstand. Scores for each test within the eSPPB assessment ranged from 0 to 1 based on a ratio of the measured value to the best possible performance. Adding across the four components gives a continuous score from 0 to 4. Although the different subcomponents of the eSPPB were designed to assess clinically relevant aspects of physical function, they are not statistically independent. A correlation matrix showing the relationships between the four subcomponents is included in supplemental Table S1.

#### Brain imaging collection, processing, and network generation

An anatomical T1-weighted 3D volumetric MPRAGE and two functional blood oxygenation level-dependent (BOLD) scans were collected on a Siemens 3 T Skyra MRI scanner with 32-channel head coil. During the resting-state fMRI scan, a fixation cross was displayed on the monitor, and for the motor imagery visualization task fMRI scan, continuous feed videos adapted from the Mobility Assessment Tool short-form (MAT-sf) [26, 27] were played on the monitor. Two versions of this motor imagery visualization, “easy” task and “hard” task were presented in the scanner. Our prior assessment of the tasks revealed that DAN community structure was strongest during the easy task in this population [10] and was thus the focus of the analyses presented here. Images were preprocessed using Statistical Parametric Mapping version 12 (SPM12, http://www.fil.ion.ucl.ac.uk/spm), FMRIB’s “topup” Software Library (FMRIB Software Library v6.0), and Advanced Normalization Tools (ANTs). Structural images were segmented based on gray and white matter using SPM12. Gray and white matter segmented images were then summed to generate a mask of brain parenchyma. Images were then masked and spatially normalized according to the Montreal Neurological Institute (MNI) template using ANTs. Functional images preprocessing included distortion correction, slice time correction, realignment, coregistration with native-space anatomical images, and warping to MNI space using transformation information from ANTs. The motion scrubbing procedure developed by Power and colleagues [28] was used to correct head motion artifacts during the scan. Signals from total white matter, total gray matter, total CSF, and the 6 rigid-body motion parameters indicated from the first realignment procedure were removed using regression. This was followed by band pass filtering (0.009–0.08 Hz) using cutoffs established in early studies of resting-state brain network organization [29]. Further details of structural and functional image processing are in the supplemental eMethods.

Networks were generated by performing voxel-wise cross-correlations on each voxel pair. The resulting matrix is a weighted brain network, where each voxel is a node and correlations between nodes are edges. An empirically determined threshold was calculated to satisfy the equation *S* = log(*N*)/log(*K*), where *S* = 2.5 and *K* is the average number of connections per node [30]. The threshold was applied to the matrix to dichotomize the data and create a final binary adjacency matrix, *Aij*, an *N* × *N* matrix (where *N* is the number of network nodes, ~ 20,000). Values at or above the threshold were set to 1 indicating the presence of a connection, and those below the threshold were set to 0.

### Community structure analyses

Modularity (*Q*) [15] was used to identify network community partitions for each study participant in each condition using a dynamic Markov process [31]. The partitioning procedure resulted in each individual participant’s brain network being divided into categorical communities. Each participant’s communities were compared to a priori templates for SMN and DAN to produce scaled inclusivity (SI) values for all brain voxels [32, 33]. The resulting maps, SMN-CS and DMN-CS, indicated the level of spatial alignment of the participant’s communities with the SMN and DAN, respectively. Values from these maps were used in the regression analyses detailed below. Further information on the scaled inclusivity analyses can be found in the supplemental Methods Section 1.3.3.

### Statistical analyses

Distance regression analyses [16] were used to assess associations between predictor variables (e.g., eSPPB components) and brain network community structure. For each combination of condition (rest and task) and network (SMN and DAN), a separate model was used. The Jaccard distance (see Methods Section 1.4) was used to quantify the distance between the SI community structure maps and served as the dependent variable. These distances were calculated with a 3-dimensional SI brain map of the network community structure for each participant. Distance (absolute distance between participants for independent variables) was computed between every subject pair to create a distance matrix for each independent variable (eSPPB component, BMI, sex, and head motion). Our prior study [18] assessed the relationships between age, sex, and race and SMN and DAN connectivity. Only sex was significant and was the only demographic used in the current analyses. All primary models included the eSPPB component*BMI interaction.

For the primary analyses presented in the manuscript, BMI was treated as a continuous measure. Although there are advantages to using the continuous measure, there can also benefits to examining BMI using traditional categories. One particular benefit is the intuitive understanding that people have of the BMI categories. We performed exploratory analyses using three BMI categories (normal weight, overweight, and obesity) to determine if results were comparable to the continuous model. Qualitatively, the continuous and categorical analyses were quite similar. The outcomes of these analyses are presented in the supplemental Results section (Tables S4 and S5 and Figures S7 and S8).

For all analyses (continuous BMI and categorical BMI), a linear statistical model with individual-level effects [16] was used to regress community structure distance against predictor variable distances. An adaptive false discovery rate was applied to correct for multiple comparisons within each subnetwork [34, 35]. For any models where the eSPPB component*BMI interaction was not significant, a reduced model was run without the interaction to assess mains effects of the eSPPB component and BMI. Further information on the statistical analyses can be found in the supplemental Methods Section 1.4.

## Results

### Population characteristics

Results from baseline measures of physical function are shown in Table 1, along with participant demographics. The average participant age was 76.4 years (SD 4.72). Among the 192 participants, 173 people self-identified as White, 18 as African American/Black, and one as Asian. Two of the White participants identified as Hispanic/Latino ethnicity. There were 84 men and 108 women. The average BMI of participants was 28.35 kg/m^2^ (5.62). The average eSPPB score was 2 out of 4 with a standard deviation of 0.523. The average balance score for the population was a 0.72 out of 1, with a standard deviation of 0.262. Scores for gait speed, complex gait speed, and lower extremity strength were all comparable, with averages of 0.49 (± 0.262), 0.40 (± 0.10), and 0.40 (± 0.119), respectively.

### eSPPB and BMI associations with SMN-CS

Results from models examining the interaction between each of the four eSPPB components and BMI for the SMN-CS at rest and during the motor imagery task are shown in Table 2. Simplified results are shown in Table 4. The eSPPB components that showed significant interactions with BMI were complex gait speed (*p* = 0.032) and lower extremity strength (*p* = 0.018). In both cases, the interaction was synergistic with higher physical function and lower BMI associated with higher spatial consistency of community structure.

**Table 2:**
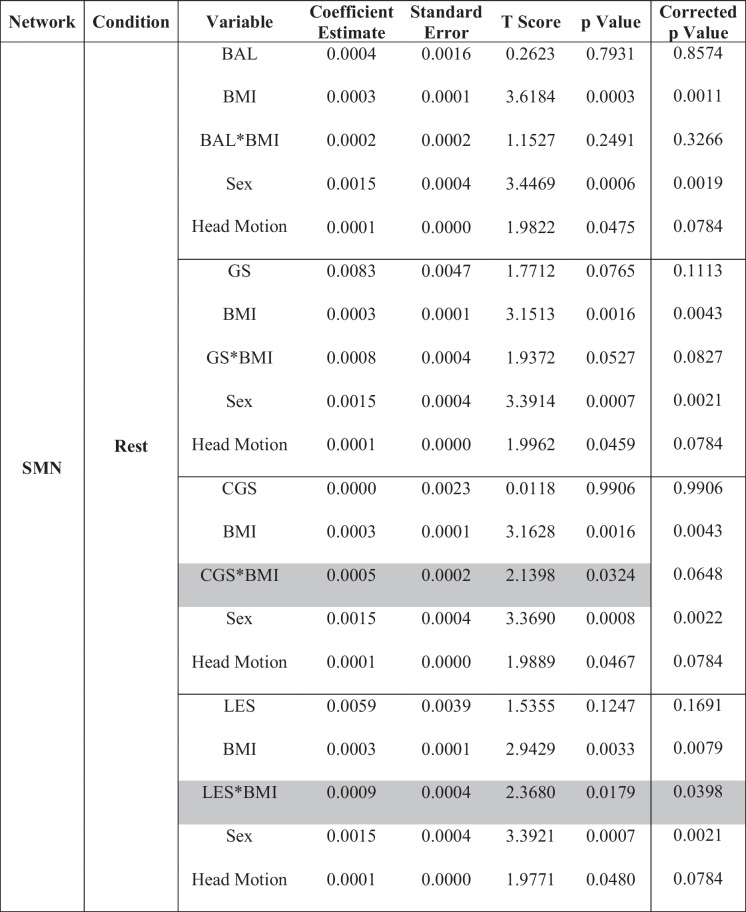

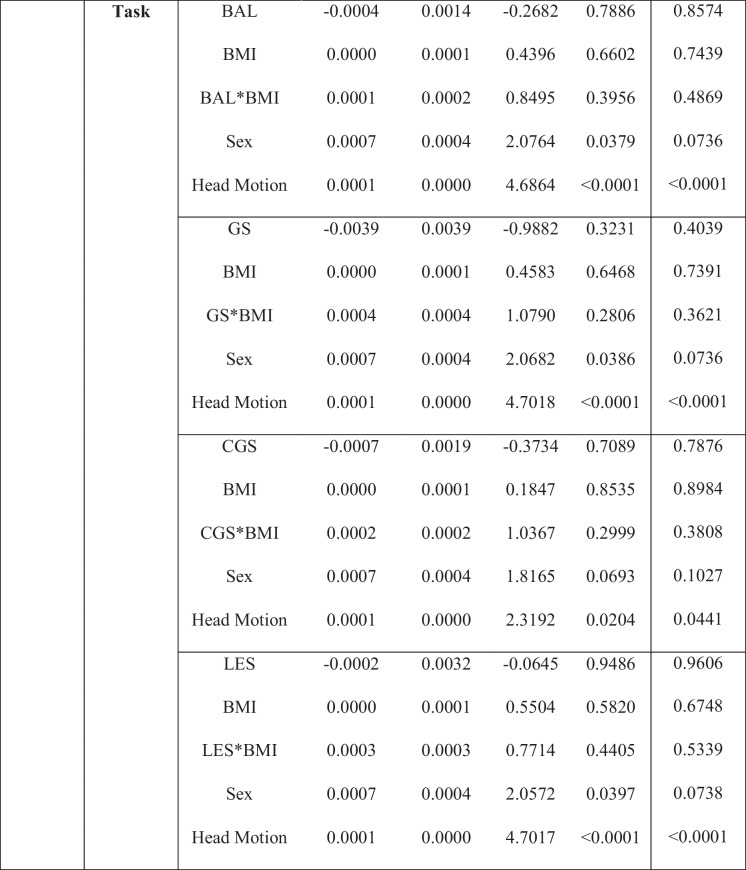
Sensory motor network: interaction models for extended Short Physical Performance Battery and body mass index

*BMI*, body mass index; *BAL*, balance; *GS*, gait speed; *CGS*, complex gait speed; *LES*, lower extremity strength

^*^Interaction. Gray shaded text indicates significant component by BMI interaction. Correction for multiple comparisons was performed using false discovery rate (FDR)

**Fig. 1:**
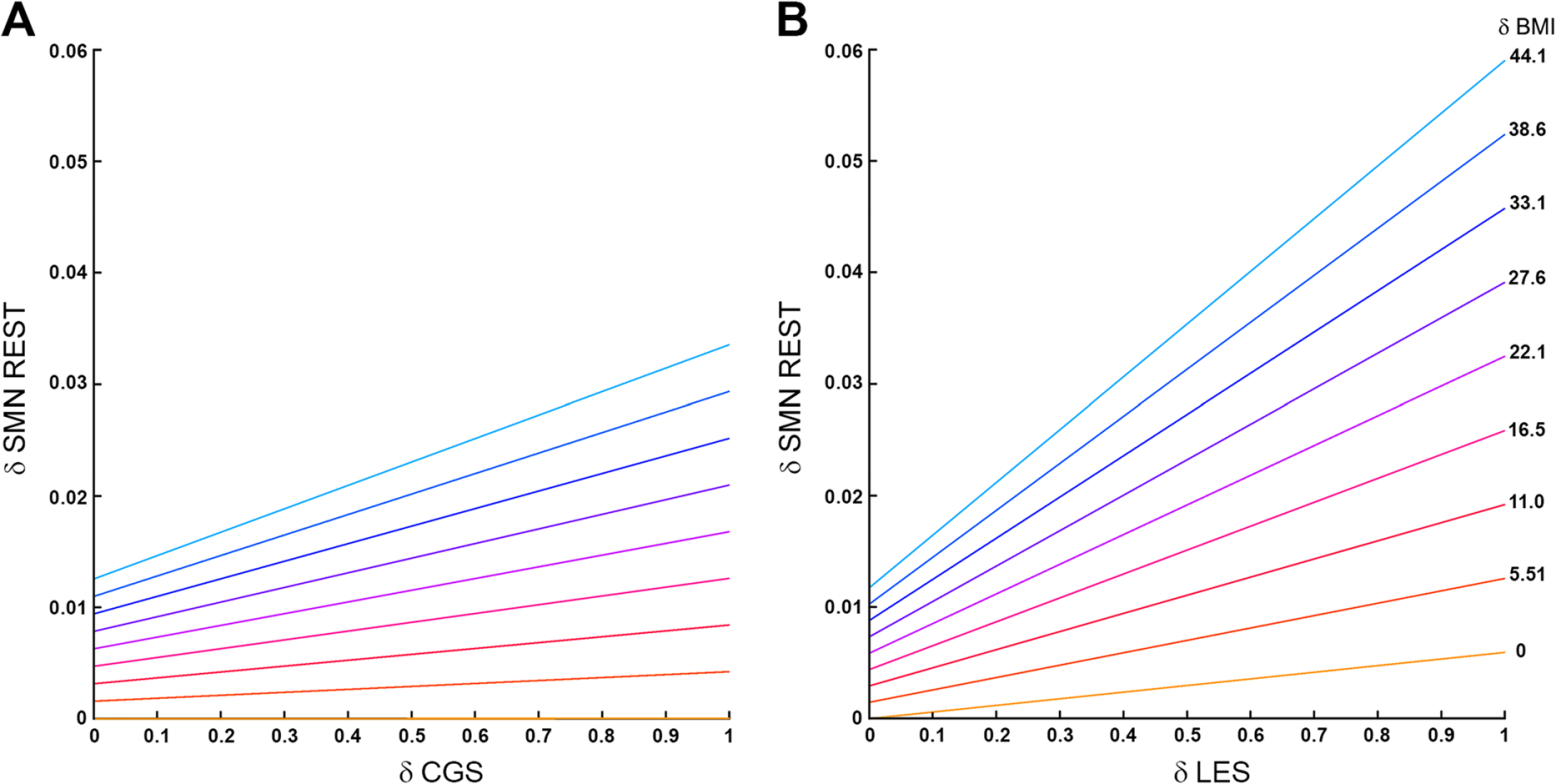
Plot of the interaction between eSPPB component complex gait speed (CGS) and lower extremity strength (LES) with BMI for SMN at rest. The color lines represent the relationships between component distances (δCGS and δLES, respectively) on the *x*-axis and community structure distances (δSMN) on the *y*-axis for ten discrete BMI distances (δBMI) that are evenly spaced. For plot **A**, the bottom yellow line shows there is essentially no relationship between δCGS and the community structure when the δBMI is 0. However, as δBMI increases the relationship between δCGS and the community structure increases. The *y*-intercepts represent the effect of δBMI when the δCGS = 0. Plot **B** shows that there is a slight positive association between δLES and the community structure when the δBMI is 0. The association increases substantially as δBMI increases, -with maximal slopes that are nearly twice that of δCGS. It is interesting to note that the effect of δBMI when the δLES = 0, the *y*-intercepts, is not larger than seen in the δCGS. Thus, the main difference between complex gait speed and lower extremity strength in the SMN is the interaction with BMI

Although there is not a simple interpretation of the estimates for the distance regression used, differences across components can be readily appreciated in the interaction plots (Fig. 1) where it is clear that the interaction between BMI and lower extremity strength is nearly twice as large as it is for complex gait speed. Spatial patterns of the associations can be seen in the brain maps of the community structure (Figure S2). No significant component by BMI interactions was seen with SMN-CS during the motor imagery task.

For each model without a significant interaction, a reduced model was run to assess main effects in the absence of the interaction. The gait speed by BMI interaction approached, but did not reach, significance at rest (*p* = 0.053). In the model without the interaction and both gait speed and BMI were each individually significant. In the balance model, BMI was significantly associated with SMN-CS at rest, but balance was not. There were no significant effects for eSPPB components or BMI in the SMN during the motor imagery task (Table 4 and Table S2).

### eSPPB and BMI associations with DAN-CS

Results from models examining the interaction between each of the four components and BMI for the DAN-CS at rest and during the motor imagery task are shown in Table 3. During the rest condition, the gait speed by BMI interaction (*p* < 0.0001) and complex gait speed by BMI interaction (*p* = 0.0013) were both significant. During the motor-imagery task, the balance by BMI (*p* = 0.0001), complex gait speed by BMI (*p* = 0.0229), and lower extremity strength by BMI (*p* = 0.003) interactions were significant.

**Table 3:**
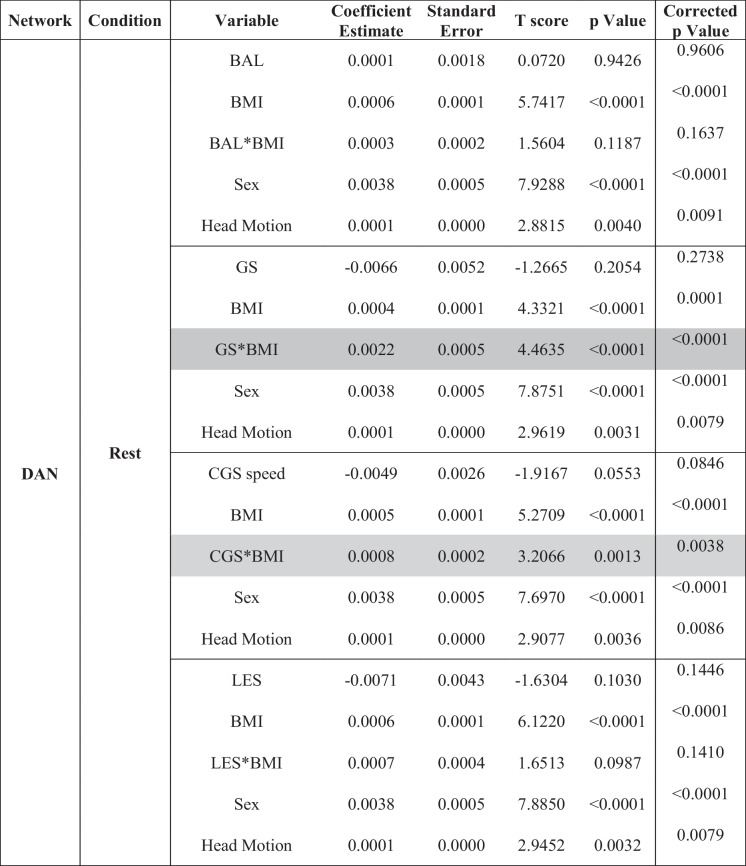

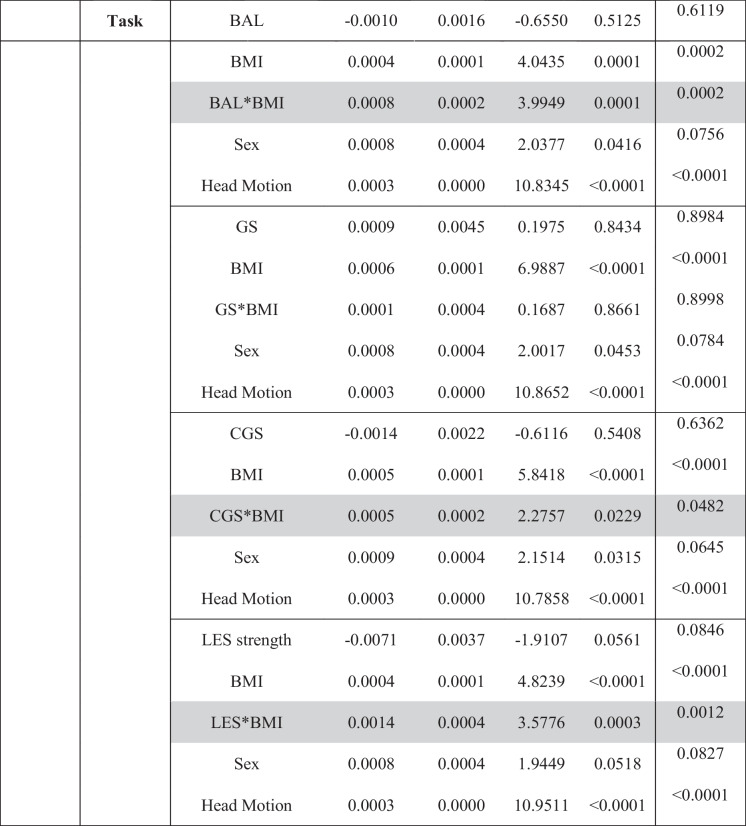
Dorsal attention network: interaction models for extended Short Physical Performance Battery and body mass index

*BMI*, body mass index; *BAL*, balance; *GS*, gait speed; *CGS*, complex gait speed; *LES*, lower extremity strength

^*^Interaction. Gray shaded text indicates significant component by BMI interaction. Correction for multiple comparisons was performed using false discovery rate (FDR)

As found in the SMN, all interactions in the DAN were synergistic with higher physical function and lower BMI being associated with higher spatial consistency of community structure. Interaction plots and maps of the community structure for the DAN are presented in Figures S3–6 in the Supplement. Reduced models that did not include the eSPPB component by BMI interactions did not identify significant main effects for any component, but BMI was significant in all models (Table 4 and Table S3).

**Table 4:**
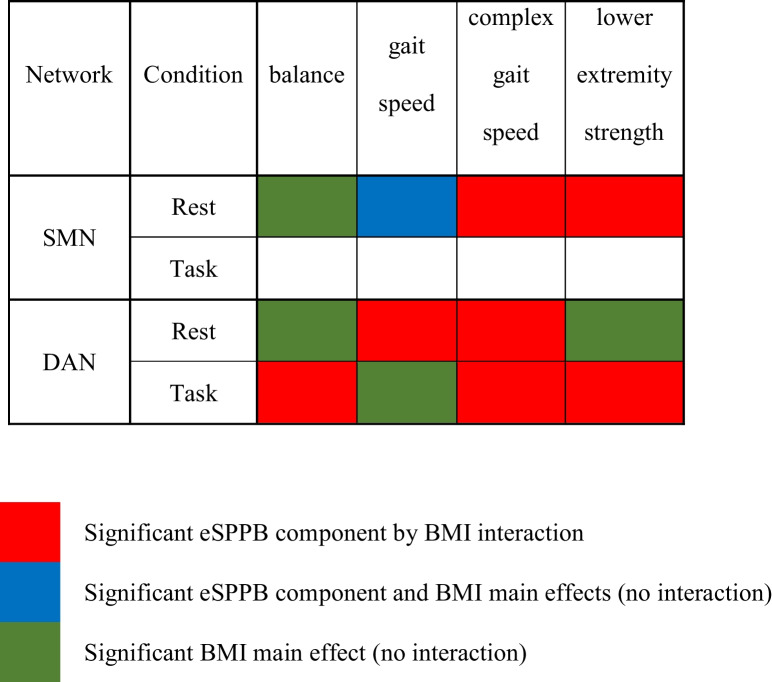
Summarized significant associations

## Discussion

This study examined the interaction between specific components of the eSPPB and BMI with functional brain network community structure in older adults. The primary hypotheses, based on our prior work [18] and the known roles of the SMN and DAN, were that gait measures (GS and CGS) and leg strength (LES) would have statistical interactions with BMI in the SMN, whereas balance (BAL) and the complex gait measure (CGS) would interact with BMI in the DAN. The main study outcomes showed that complex gait speed and lower extremity strength both exhibited statistical interactions with BMI in the SMN, only at rest. After adjusting for multiple comparisons, the role of chance in the complex gait speed finding could not be ruled out. There were no significant associations in the SMN during the motor imagery task. It was also found that gait speed and complex gait speed statistically interacted with BMI in the DAN at rest. During the task, balance, complex gait speed, and lower extremity strength all significantly interacted with BMI. All significant relationships were such that higher performance on components of the eSPPB and/or lower BMI were associated with higher spatial consistency in the SMN or DAN community structure.

These results are novel, largely support our initial hypotheses, and are consistent with current knowledge about the brain regions encompassed by these brain networks. The SMN is known to produce the motor commands necessary for movement. Thus, it is not surprising that leg strength and gait speed were associated with the integrity of this network. The integrity of SMN connectivity has been shown to be higher in individuals with better mobility by our group [18, 36] and others [7, 12]. The statistical interactions that were found support the idea that BMI and the subscale measures amplify each other’s association with the brain network. For example, for lower extremity strength, the findings suggest that individuals with low leg strength have degraded SMN but those individuals that also have a high BMI have even greater degradation. If someone has weak legs and is not able to ambulate well, this could lead to disuse of the SMN and loss of network integrity. If they have a high BMI that is only going to amplify their mobility limitations and result in even great disuse of the SMN. Having identified these neural correlates of lower extremity strength, longitudinal studies should determine if SMN-CS is predictive of future age-related mobility declines given the important of leg strength for mobility.

The current study extends our prior finding that the interaction between eSPPB (total score) and BMI in the SMN was limited to the resting state [18]. Although traditional fMRI studies have shown higher levels of activity, or BOLD signal, during the motor imagery [37, 38], this activation is not universal throughout the network. Rather, portions of the sensorimotor cortex devoted to the legs were active during imagined walking. Community structure analyses, as used here, identify circuits that are highly interconnected, rather than strictly showing areas of activation. Prior studies have shown that the SMN is highly interconnected at rest [32, 39–41]. This high interconnectivity is indicative of large-scale synchrony throughout the SMN with all regions sharing common information. It has also been shown with magnetoencephalography (MEG) that resting SMN synchrony is disrupted and becomes desynchronized immediately before movements [42] and during motor imagery [11]. Thus, it is not surprising that the current findings were mainly at rest when the SMN is most likely to be synchronized.

The DAN is typically involved in higher order spatial attention and orientation, linking salient stimuli to motor responses [43, 44]. This functionality is the basis for our hypothesis that balance and complex gait would be associated with DAN-CS. Recent work suggests that DAN connectivity is associated with gait variability rather than gait speed [45]. Our findings for resting state supported our hypothesis implicating gait in the integrity of DAN-CS, but there were no balance associations in the DAN at rest. Our hypothesis that balance and complex gait would be associated with DAN was supported for the motor imagery task. It has been reported that the DAN is active and important in motor imagery [44, 46]. Thus, it may be that associations between the measures of physical function and DAN-CS may be best assessed when the DAN is engaged in a task directly relevant to its role in locomotion. The association between lower extremity strength and DAN-CS during the task is likely related to the fact that spatial attention is an embodied process [22] and that lower extremity strength is highly relevant to the movements shown in the MAT-sf video used during the motor imagery task.

Although the primary objective of this work was to relate various aspects of physical function to brain network organization, our analyses included an interaction with BMI as we found this in our prior work. The average BMI score of participants was 28.4 (Table 1), and 59 participants qualified as obese (i.e., BMI ≥ 30). For the main outcomes, BMI was treated as a continuous variable. However, supplementary analyses were run for specific BMI categories (normal weight, overweight, obesity) largely replicating the main study outcomes. An interesting finding here was that the BMI association was highly consistent regardless of the eSPPB component being investigated. In addition, when the interaction was not significant, BMI always had a significant main effect except in the SMN during the task. It has been established that BMI is associated with mobility limitations, higher risk of mobility disability, and lower SPPB scores [47–49]. Obesity is associated with difficulties rising from a chair and other tasks that fight gravity. It is possible that those individuals with obesity stand less often, which may lead to less engagement and reinforcement of the relevant networks. Obesity is also associated with reductions in brain gray and white matter volume [50, 51] and white matter integrity [50, 52, 53]. Since the brain white matter contains the fibers that interconnect brain regions, damage to white matter can disrupt network connectivity. Given the associations that obesity has with pathological brain changes, it is not surprising that we found elevated BMI to be associated with reduced functional network integrity.

Although our analyses were designed to discover neural associations with components of the eSPPB and BMI, the correlative nature of these analyses does not allow for causal interpretations. It is possible that low physical activity due to a variety of neurocognitive or physical limitations (such as depression or osteoarthritis) results in a disuse reduction in the integrity of these networks. Similarly, obesity can impair mobility and physical function, and this would lead to SMN and DAN disuse. In such cases, interventions could be targeted at increasing physical activity and reducing sedentary behavior [54]. On the other hand, it is possible that disruption of the SMN and DAN integrity due to brain-based pathology (such as cerebrovascular disease) could lead to decreased physical function. If degradation of brain network integrity is driving the decline in physical function, neuromodulatory treatments, such as transcranial magnetic stimulation (TMS) or transcranial direct current stimulation (tDCS), may prove useful. In fact, it has been shown that tDCS applied to the DAN modulates gait variability [45]. Another possibility is that the relationships between physical function and brain networks exhibit circular causality, as is thought to occur in complex biological systems [55, 56] including the brain [57]. In such a case, whichever came first may not matter as either degraded network organization or impairments in physical function can cause the other, resulting in spiraling declines. This is an important topic for future research as circular causality would allow for interventions that are either brain- or behavior-based (or both) regardless of the initial instigating factor. Thus, an optimist perspective would be that understanding brain-body interactions may help us turn spiraling declines into upward spirals.

This study is not without limitations. As noted above, the cross-sectional study design does not allow for the identification of causal processes. However, BNET does have a longitudinal component that will be used in future analyses to determine if baseline brain networks predict decline in physical function, or vice versa. Although this is a relatively large study sample, the population included community-dwelling, relatively healthy/high-functioning older adults. The type of community structure analyses used are novel and it is unknown if differences in various methodological choices could impact the findings. We used voxel-wise analyses and our own templates for the SMN and DAN. These methods were specifically chosen to match our prior study examining eSPPB and community structure. However, future work should consider examining replicability using alternative methods of analyzing brain network community structure such as applying one of the growing number of parcellation schemes [58–61]. Given that our work was focused on two specific subnetworks (SMN and DAN) rather than exploring the entire brain, it will also be important to extend analyses to other brain subnetworks, such as the default mode and ventral attention networks to determine the specificity of these findings. Future studies will also need to determine if the relationships observed here are found in more diverse samples or in those with poor health or cognition. Although our findings with BMI were quite strong and consistent, BMI is not a universally accepted measure, and there is growing interest in using body fat measures rather than BMI.

## Conclusions

This study shows clear evidence that specific components of physical function interact with BMI within the SMN and DAN. In the SMN, the associations were for gait (gait speed and complex gait speed) and strength (lower extremity strength) measures, but only during rest. In the DAN, the associations differed depending on condition. At rest, the DAN associations were for gait measures (gait speed and complex gait speed). During the motor imagery task, the associations included balance and strength measures as well. Gait speed and balance are two objective measures that can act as primary indicators of functional mobility [20, 21]. Slower walking pace has been shown to be predictive of disability, cognitive impairment, mortality, and falls in older adults [20]. Balance is most obviously associated with fall frequency and is also associated with loss of independence, blood pressure disorders, and certain medications [62]. Results from this study expand our understanding of how different components of physical function, such as balance or gait speed, are associated with network structure. Gaining a greater mechanistic understanding of the associations between low physical function and brain physiology may lead to the implementation of new and/or personalized treatments based on the specific limitation in physical function.

### Supplementary Information

Below is the link to the electronic supplementary material.

Supplementary file1 (PDF 1.00 MB)

## Data Availability

Data can be made available upon request to the authors with appropriate Institutional Review Board approval and data use agreements.
