# Supplementary material for: Association of physical function with connectivity in the sensorimotor and dorsal attention networks: why examining specific components of physical function matters
Source: GeroScience. 2024 Jul 5;46(5):4987–5002. doi: 10.1007/s11357-024-01251-8 (PMC11336134; doi:10.1007/s11357-024-01251-8)
Supplement: Supplementary file 1 — Supplementary file1 (PDF 1.00 MB) [file 11357_2024_1251_MOESM1_ESM.pdf]

1. Supplementary Methods

1.1. Brain Imaging

All images were collected on a Siemens 3T Skyra MRI Scanner with a 32-channel head coil. Scan sessions lasted about one hour. Anatomical images were collected using a T1-weighted 3D volumetric MPRAGE (TR = 2000 msec; TE = 2.98 msec; number of slices = 192; slice thickness = 1.0 mm; voxel dimensions = 1.0 x 1.0 x 1.0 mm; FOV = 256 mm; scan duration = 312 sec). Blood oxygenation level-dependent imaging (BOLD) (1) were used to collect functional MRI (fMRI) data at rest and during motor imagery tasks (TR = 2000 msec; TE = 25 ms; number of slides = 35; slice thickness = 5.0 mm; voxel dimensions= 4.0 x 4.0 x 5.0 mm; FOV = 256mm). Additional imaging collected but not used for these analyses included Fluid-attenuated inversion recovery (FLAIR), diffusion tensor imaging (DTI), Perfusion imaging and T2-Relaxation-Under-Spin-Tagging (TRUST) were collected but not used.

1.2. fMRI Protocol

Participants completed a resting state scan and two motor imagery task scans using video animations. Resting scans were performed first. Resting scans included 230 images (scan duration = 460 s). The order of motor imagery tasks was randomized. Motor imagery tasks had 130 images (scan duration = 260 s). BOLD Scans were collected parallel to the anterior commissure-posterior commissure (AC-PC) using multi-spire gradient-echo plana imaging (EPI). Participants laid supine in the MRI scanner with visibility of a large monitor at the head-end of the scanner, viewed through a mirror. During the resting state scan, a fixation cross was displayed on the monitor. During motor imagery task scans, continuous feed videos were played on the monitor. Videos were adapted from the Mobility Assessment Tool short form, MAT-sf (2, 3) and were played using a standard media player. Videos showed an avatar performing “easy” and “hard” mobility tasks. Tasks were given these designations based on prior work in older adults (3). Comparisons between conditions have been reported in a subsampled of the BNET study (4) and based on those results, the current study only used the “easy” task. Participants were

given instructions and completed a visual imagery practice session prior to entering the scanner. During the practice visual imagery session, participants viewed shortened videos and were instructed to imagine themselves as the avatar and told that their active involvement and engagement in the task is crucial to the validity of the experiment. More details about the task instructions, training, and videos have been reported (4).

1.3. Image Analysis

1.3.1. High-resolution anatomical image

Structural image segmentation was completed using Statistical Parametric Mapping version 12 http://www.fil.ion.ucl.ac.uk/spm. Segmented gray and white matter images were summed. Any voxel with ≥0.5 probably value was retained as a mask of brain parenchyma with non-brain tissue and cerebral spinal fluid (CSF) excluded. Structural images were masked, visually inspected, and manually cleaned to remove remaining extra-parenchymal tissues using MRIcron software (5). To ensure full-brain coverage, manual checking was performed by two observers. Masked and cleaned T1-weighted images were spatially normalized to the Montreal Neurological Institute (MNI) template using Advanced Normalization Tools (ANTs) (6).

1.3.2. Functional image analyses

FMRIB's "Topup" Software Library (FMRIB Software Library v6.0) (7) was used for distortion correction. For signal normalization purposes, the first 10 volumes of BOLD images were dropped. SPM12 was used to correct slice time and realign functional images. BOLD images were coregistered to native-space anatomical imaged and warped to MNI space using the ANT-derived transformation. Power's motion scrubbing was used for motion correction to remove volumes containing excessive movement (> 0.5 mm FD) and excessive signal change (> 0.5 DV_{GM}) (8). An average of 7.9±13.9

volumes in the rest condition and 8.8±12.9 volumes in the task condition were dropped across participants. Data were band-pass filtered (0.009-0.08 Hz) in order to account for low-frequency drift and physiological noise in images. Confounding signals (white matter, gray matter, CSF, and 6 rigid-body motion parameters generated from realignment) were regressed out from filtered data.

1.3.3. Community structure analyses

A network community is a group of nodes that are more connected with one another than other nodes (9). The binary network for each participant was partitioned into individual communities (neighborhoods) with each voxel (node) assigned to a single community. Network partitioning optimization was created using modularity (Q) (10). A dynamic Markov process (11) was used to identify network partition that maximized Q. Because of the stochastic component in the modularity algorithm, it was run 100 times and the partition associated with the highest Q value was used. Partitioning resulted in the sectioning of each participant's brain network into categorical communities. Each node is assigned to its correct community and data can be mapped into brain space to visualize the spatial distribution of network communities.

For group analyses, spatial alignment of communities is compared across participants to create a scaled Inclusivity (SI), which is computed to quantify spatial similarity across participants (12). To determine spatial similarity for specific networks, *a priori* templates are used as comparators when calculated SI. SMN and DAN templates were generated using resting-state brain network data in 22 normal adults from a prior study (13). SI values range from 0 to 1; with 1 representing a perfect spatial alignment of template community with communities across all participants for that community. This is a hypothetical value. In practice, nodes are assigned values less than 1, with specific value depending on spatial variability across subjects (14). High SI values indicate a stable network community across

subjects that occupies the same or similar brain regions across people. Low SI values indicate lower spatial consistency of a community across people. SI values can be used to identify differences in community organization between populations. Relative magnitudes of SI values between groups or conditions are more useful than absolute scores for interpretations. Because analyses were performed on distances, not raw variables, it isn't possible to visualize brain maps of SI residuals from the regression. Community structure maps are therefore generated using average voxel-wise SI values for each condition (e.g. DAN at rest) for upper and lower tertiles of the independent variable of interest. All brain images are displayed using MRIcro software (https://people.cas.sc.edu/rorden/mricro/).

1.4. Statistical Analyses

Analyses used a distance regression approach to examine associations between predictor variables and brain network community structure. This method was developed to assess relationships between brain networks and continuous and/or categorical variables while controlling for confounding and or nuisance variables. The original publication contains statistical details of this method (15). The input for each study participant was a continuous variable SI map of community structure and a list of continuous and or categorical variables. Distance metrics are used to compare brain network data between subject pairs, and the generated values are regressed against absolute differences in other model variables for the same subject pairs. Distances are computed for every subject to generate a distance matrix for each variable included in the model. SI maps were compared using the Jaccardized Czekanowski similarity index [15]. Because it is a similarity index, rather than a distance index, the Jaccard distance (1-Jaccard index) was used for all analyses. Estimation and inference for the regression models were performed using an F test with individual level effects (ILE).

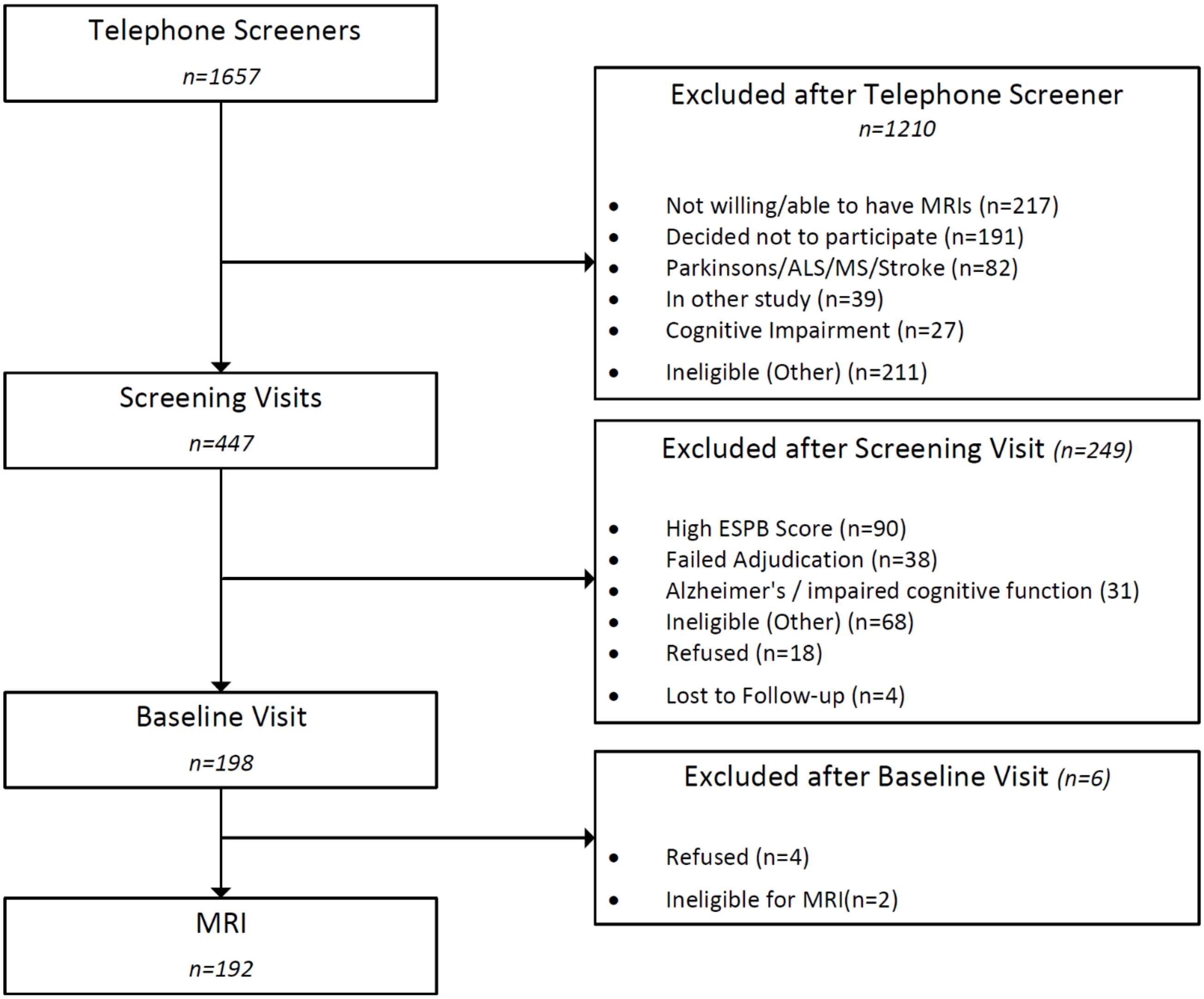

Figure S1: A Strengthening the Reporting of Observational Studies in Epidemiology (STROBE) diagram for participant recruitment/retention through the baseline MRI visit of BNET. Longitudinal tracking is not presented as those data were not used in the current study.

2. Supplementary Results

Table S1: Associations between eSPPB subcomponents

	BAL	GS	LES	CGS
BAL	1	0.41	0.24	0.51
GS		1	0.44	0.62
LES			1	0.45
CGS				1

The statistical association between the esppb subcomponents was assessed with a Person's correlation. The correlation matrix (r-values) below shows that GS and CGS exhibited the highest correlation while BAL and LES had the lowest.

Table S2: SMN-CS main effects

**Network**	**Condition**	**Variable**	**Estimate**	**SE**	**T Score**	**pValue**
**SMN**	**Rest**	BAL	0.0017	0.0012	1.4453	0.1484
		BMI	0.0004	0.0001	5.7731	0.0000
		Sex	0.0015	0.0004	3.4432	0.0006
		Head Motion	0.0001	0.0000	1.9775	0.0480
		GS	0.0138	0.0037	3.6964	0.0002
		BMI	0.0004	0.0001	5.3929	0.0000

		Sex	0.0015	0.0004	3.4030	0.0007
		Head Motion	0.0001	0.0000	1.9707	0.0488
	**Task**	BAL	0.0004	0.0010	0.4314	0.6662
		BMI	0.0001	0.0001	1.3142	0.1888
		Sex	0.0007	0.0004	2.0737	0.0381
		Head Motion	0.0001	0.0000	4.6853	0.0000
		GS	-0.0013	0.0031	-0.4225	0.6727
		BMI	0.0001	0.0001	1.3826	0.1668
		Sex	0.0007	0.0004	2.0749	0.0380
		Head Motion	0.0001	0.0000	4.7189	0.0000
		CGS	0.0005	0.0014	0.3783	0.7052
		BMI	0.0001	0.0001	1.0119	0.3116
		Sex	0.0007	0.0004	1.8303	0.0672
		Head Motion	0.0001	0.0000	4.8128	0.0000
		LES	0.0012	0.0027	0.4410	0.6592
		BMI	0.0001	0.0001	1.3052	0.1918
		Sex	0.0007	0.0004	2.0708	0.0384
		Head Motion	0.0001	0.0000	4.6950	0.0000

Note. SMN-CS – sensorimotor network community structure, SE – standard error, BMI – body mass index, BAL – balance, GS – gait speed, CGS – complex gait speed, and LES – lower extremity strength

Table S3: DAN-CS main effects

Network	Condition	Variable	Estimate	SE	T Score	pValue
**DAN**	**Rest**	BAL	0.0021	0.0013	1.5676	0.1170
		BMI	0.0007	0.0001	8.9288	0.0000
		Sex	0.0038	0.0005	7.9236	0.0000
		Head Motion	0.0001	0.0000	2.8750	0.0040
		LES	-0.0031	0.0036	-0.8544	0.3929
		BMI	0.0007	0.0001	9.0736	0.0000
		Sex	0.0038	0.0005	7.9143	0.0000
		Head Motion	0.0001	0.0000	2.9431	0.0033
	**Task**	GS	0.0013	0.0036	0.3755	0.7073
		BMI	0.0006	0.0001	8.8643	0.0000
		Sex	0.0008	0.0004	2.0028	0.0452
		Head Motion	0.0003	0.0000	10.8693	0.0000

Note. DAN-CS – dorsal attention network community structure, SE – standard error, BMI – body mass index, BAL – balance, GS – gait speed, and LES – lower extremity strength

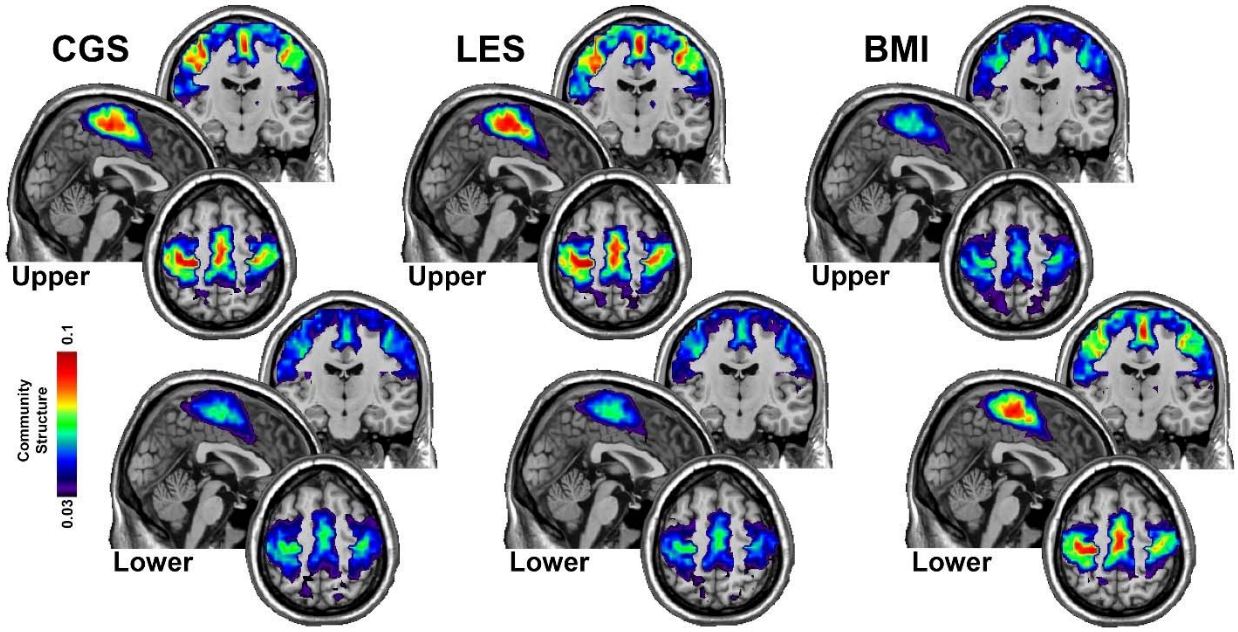

Figure S2: Community structure maps for SMN during the rest condition for the upper and lower tertiles for complex gait speed (CGS), lower extremity strength (LES), and body mass index (BMI). For CGS and LES, the upper tertiles had greater community spatial overlap across participants compared to the lower tertiles. The opposite relationship was true for BMI, having higher community structure in the lower vs. upper tertile. Warmer colors indicate higher community structure, or an area that is more frequently a part of the brain community across participants. BMI – body mass index, CGS – complex gait speed, and LES – lower extremity strength.

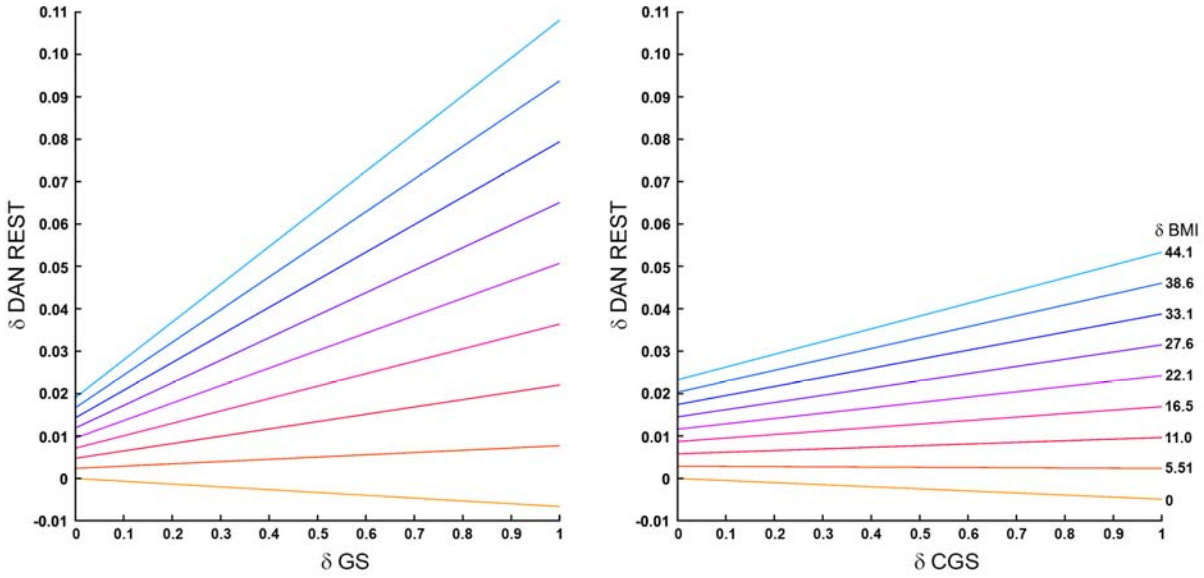

Figure S3: Plot of the interaction between eSPPB components gait speed (GS) and complex gait speed (CGS) with BMI for DAN at rest. The color lines represent the relationships between component distances (δGS, and δCGS) on the x-axis and community structure distances (δDAN) on the y-axis for ten discrete BMI distances (δBMI) that are evenly spaced. The yellow lines represent the effects of δGS, and δCGS when δBMI=0. The y-intercepts represent the effect of δBMI when the δGS and δCGS each equal 0.

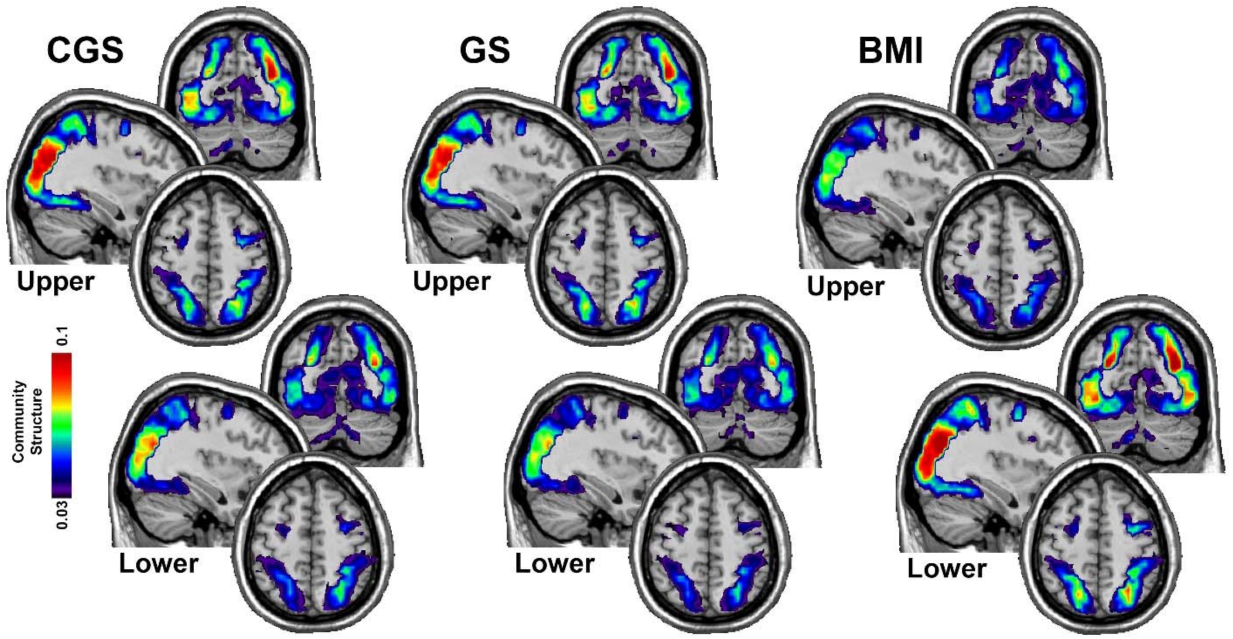

Figure S4: Community structure maps for DAN during the rest condition for the upper and lower tertiles of complex gait speed (CGS), gait speed (GS), and body mass index (BMI).CGS, GS, and BMI. For CGS and GS, the upper tertiles had greater community spatial overlap across participants compared to the lower tertiles. The opposite relationship was true for BMI, having higher community structure in the lower vs. upper tertile. The color bar indicates the same measures as in eFigure 2. BMI – body mass index, GS – gait speed, and CGS – complex gait speed

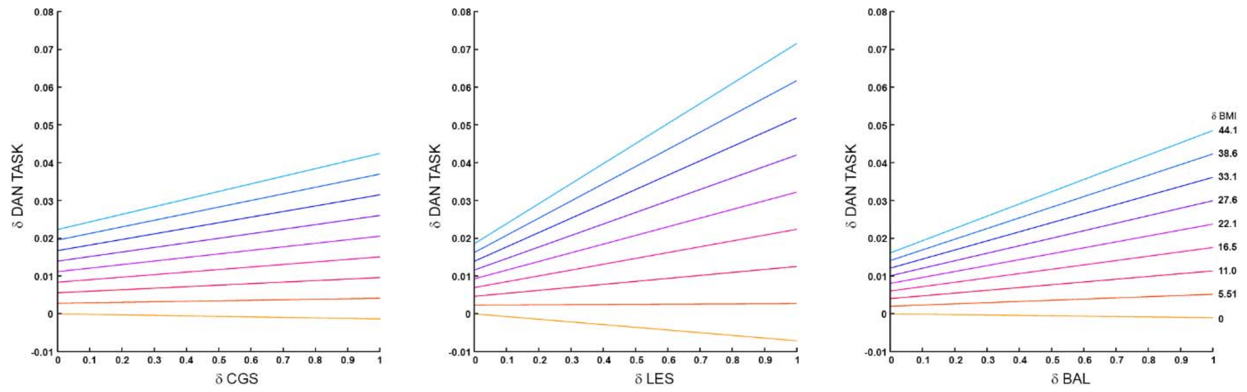

Figure S5: Plot of the interaction between eSPPB components complex gait speed (CGS), lower extremity strength (LES), and balance (BAL) with BMI for DAN during the motor imagery task. The y-axis, x-axis and relationship as described by the colored lines is the same as in eFigure 3.

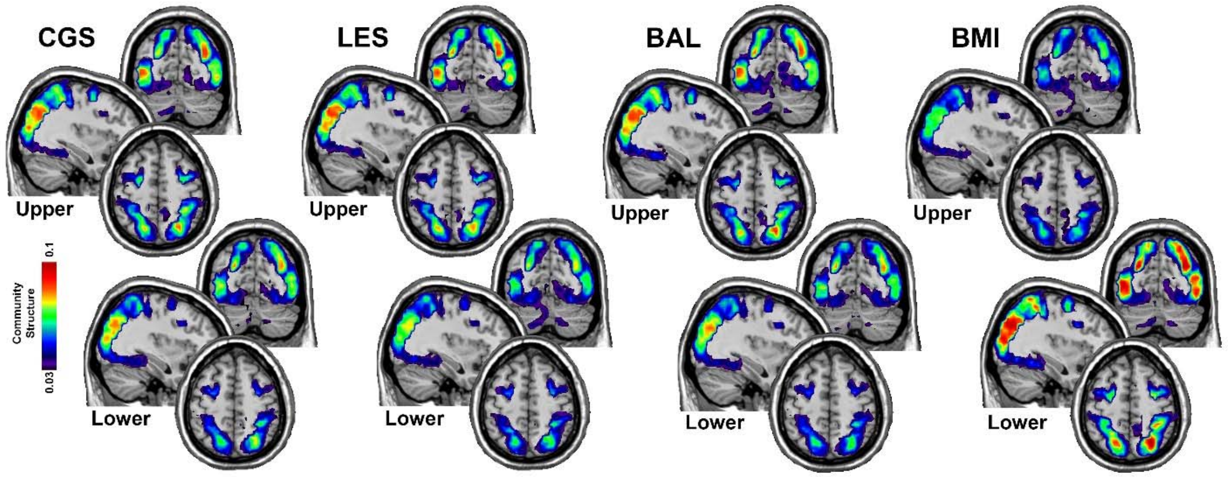

Figure S6: Community structure maps for DAN during the motor imagery task for the upper and lower tertiles of complex gait speed (CGS), lower extremity strength (LES), balance (BAL) and body mass index (BMI). BMI, CGS, LES and BAL. For BMI, the lower tertile had greater community spatial overlap across participants. The opposite relationship was true for the subscale measures CGS, LES, and BA, each having higher community structure in the upper tertile. The color bar indicates the same measures as in eFigure 2. BMI – body mass index, BAL – balance, CGS – complex gait speed, and LES – lower extremity strength

Categorical BMI analyses

Exploratory analyses were performed using BMI as a categorical variable. Individuals were assigned the following categories: normal weight (BMI = 18.5-24.9), overweight (BMI = 25.0 – 29.9) or obesity (BMI $\geq$30). Two participants were categorized as underweight (BMI < 18.5) and were exclude form the analyses. There were 52, 77, and 61 participants in the normal weight, overweight, and obesity categories. Given the ordinal nature of the BMI categories and the hypothesis that there will be linear association between BMI and the brain networks, the categories were coded as 0, 1, and 2. In the distance regression model this resulted in there being a distance of 1 between normal weight and overweight and between overweight and obesity. There was a distance of 2 between normal weight and obesity.

The findings presented below in Tables S4 and S5 revealed very similar results to the analyses using BMI as a continuous variable. The estimates for all of the significant interactions were in the same direction across both analyses. Note that the absolute magnitude of the estimates for the interaction between BMI and the eSPPB categories cannot be directly compared between the continuous and categorical analyses. The overall take way from the two analyses were quite similar (Table S6). A notable difference was that the two significant leg strength interactions observed in the continuous model were no longer significant in the categorical model. This suggests that variability within the BMI categories may be important for that interaction. Another difference was that the BMI*gait speed interaction was significant for DAN-Task in the categorical model but not in the continuous model. Further exploration of the differences between the two the continuous and categorical BMI associations is warranted. Figure S7 shows the SMN and DAN community structure for the 3 groups. It is clear that the community structure declines in a step-wise fashion from normal weight to overweight to obesity.

Table S4: SMN-CS Categorical BMI Results

Network	Condition	Variable	Coefficient Estimate	Standard Error	T Score	p Value
SMN	Rest	BAL	0.0013	0.0016	0.8299	0.4066
		BMI	0.0019	0.0005	3.6087	0.0003
		BAL*BMI	0.0002	0.0013	0.1183	0.9058
		Sex	0.0017	0.0004	4.0429	0.0001
		Head Motion	0.0001	0.0000	2.0323	0.0421
		GS	0.0080	0.0049	1.6385	0.1013
		BMI	0.0013	0.0005	2.6265	0.0086
		GS*BMI	0.0042	0.0035	1.2048	0.2283
		Sex	0.0017	0.0004	3.9984	0.0001
		Head Motion	0.0001	0.0000	2.0350	0.0419
		CGS	-0.0015	0.0022	-0.6652	0.5059
		BMI	0.0007	0.0005	1.4401	0.1499
		CGS*BMI	0.0055	0.0018	3.1409	0.0017
		Sex	0.0017	0.0004	3.9781	0.0001
		Head Motion	0.0001	0.0000	2.0180	0.0436
		LES	0.0087	0.0039	2.2194	0.0265
		BMI	0.0017	0.0005	3.4646	0.0005
		LES*BMI	0.0009	0.0029	0.3147	0.7530
		Sex	0.0017	0.0004	4.0313	0.0001
		Head Motion	0.0001	0.0000	2.0274	0.0426
	Task	BAL	-0.0009	0.0014	-0.6343	0.5259

		BMI	-0.0001	0.0004	-0.2186	0.8270
		BAL*BMI	0.0015	0.0011	1.2884	0.1976
		Sex	0.0008	0.0004	2.1569	0.0310
		Head Motion	0.0001	0.0000	4.5774	<0.0001
		GS	-0.0037	0.0042	-0.8949	0.3709
		BMI	0.0001	0.0004	0.1296	0.8969
		GS*BMI	0.0027	0.0030	0.8976	0.3694
		Sex	0.0008	0.0004	2.1554	0.0311
		Head Motion	0.0001	0.0000	4.6061	<0.0001
		CGS	-0.0019	0.0019	-0.9776	0.3283
		BMI	-0.0003	0.0004	-0.6872	0.4920
		CGS*BMI	0.0028	0.0015	1.8991	0.0576
		Sex	0.0007	0.0004	1.9130	0.0558
		Head Motion	0.0001	0.0000	4.6952	<0.0001
		LES	0.0007	0.0034	0.2029	0.8392
		BMI	0.0003	0.0004	0.5980	0.5498
		LES*BMI	0.0006	0.0024	0.2443	0.8070
		Sex	0.0008	0.0004	2.1500	0.0316
		Head Motion	0.0001	0.0000	4.5895	<0.0001

Note. BMI = body mass index as a categorical variable (normal weight, overweight, and obesity), BAL = balance, GS = gait speed, CGS = complex gait speed, LES = lower extremity strength and *=interaction. Gray shaded text indicates significant component by BMI interaction.

Table S5: DAN-CS Categorical BMI Results

Network	Condition	Variable	Coefficient Estimate	Standard Error	T score	p Value
DAN	Rest	BAL	0.0000	0.0018	0.0225	0.9821
		BMI	0.0022	0.0006	3.7084	0.0002
		BAL*BMI	0.0021	0.0015	1.3988	0.1619
		Sex	0.0037	0.0005	7.6800	<0.0001
		Head Motion	0.0001	0.0000	2.8880	0.0039
		GS	-0.0126	0.0055	-2.2664	0.0234
		BMI	0.0003	0.0006	0.4563	0.6482
		GS*BMI	0.0225	0.0040	5.6101	<0.0001
		Sex	0.0037	0.0005	7.6517	<0.0001
		Head Motion	0.0001	0.0000	2.9450	0.0032
		CGS speed	-0.0052	0.0025	-2.0562	0.0398
		BMI	0.0013	0.0006	2.2895	0.0221
		CGS*BMI	0.0072	0.0020	3.6278	0.0003
		Sex	0.0037	0.0005	7.4698	<0.0001
		Head Motion	0.0001	0.0000	2.8793	0.0040
		LES	-0.0035	0.0045	-0.7756	0.4380
		BMI	0.0030	0.0006	5.2187	<0.0001
		LES*BMI	-0.0009	0.0033	-0.2844	0.7761
		Sex	0.0037	0.0005	7.6697	<0.0001
		Head Motion	0.0001	0.0000	2.9329	0.0034
	Task	BAL	-0.0007	0.0016	-0.4376	0.6617

		BMI	0.0010	0.0005	2.0887	0.0367
		BAL*BMI	0.0044	0.0013	3.3526	0.0008
		Sex	0.0008	0.0004	1.8359	0.0664
		Head Motion	0.0003	0.0000	10.4041	<0.0001
		GS	-0.0062	0.0048	-1.3141	0.1888
		BMI	0.0014	0.0005	2.8949	0.0038
		GS*BMI	0.0083	0.0034	2.4298	0.0151
		Sex	0.0008	0.0004	1.8057	0.0710
		Head Motion	0.0003	0.0000	10.4364	<0.0001
		CGS	-0.0016	0.0022	-0.7225	0.4700
		BMI	0.0014	0.0005	2.9247	0.0035
		CGS*BMI	0.0046	0.0017	2.6671	0.0077
		Sex	0.0008	0.0004	1.9664	0.0493
		Head Motion	0.0003	0.0000	10.3692	<0.0001
		LES strength	-0.0034	0.0038	-0.8874	0.3749
		BMI	0.0019	0.0005	3.9554	0.0001
		LES*BMI	0.0034	0.0028	1.2238	0.2211
		Sex	0.0008	0.0004	1.8115	0.0701
		Head Motion	0.0003	0.0000	10.5039	<0.0001

Note. BMI = body mass index as a categorical variable (normal weight, overweight, and obesity), BAL = balance, GS = gait speed, CGS = complex gait speed, LES = lower extremity strength and *=interaction. Gray shaded text indicates significant component by BMI interaction.

Table S6: Summarized Significant Associations

Network	Condition	balance	gait speed	complex gait speed	lower extremity strength
SMN	Rest	[green]	[blue]	[red]	[blue]
	Task				
DAN	Rest	[green]	[red]	[red]	[green]
	Task	[red]	[red]	[red]	[green]

- [red] Significant eSPPB component by BMI interaction
- [blue] Significant eSPPB component and BMI main effects (no interaction)
- [green] Significant BMI main effect (no interaction)

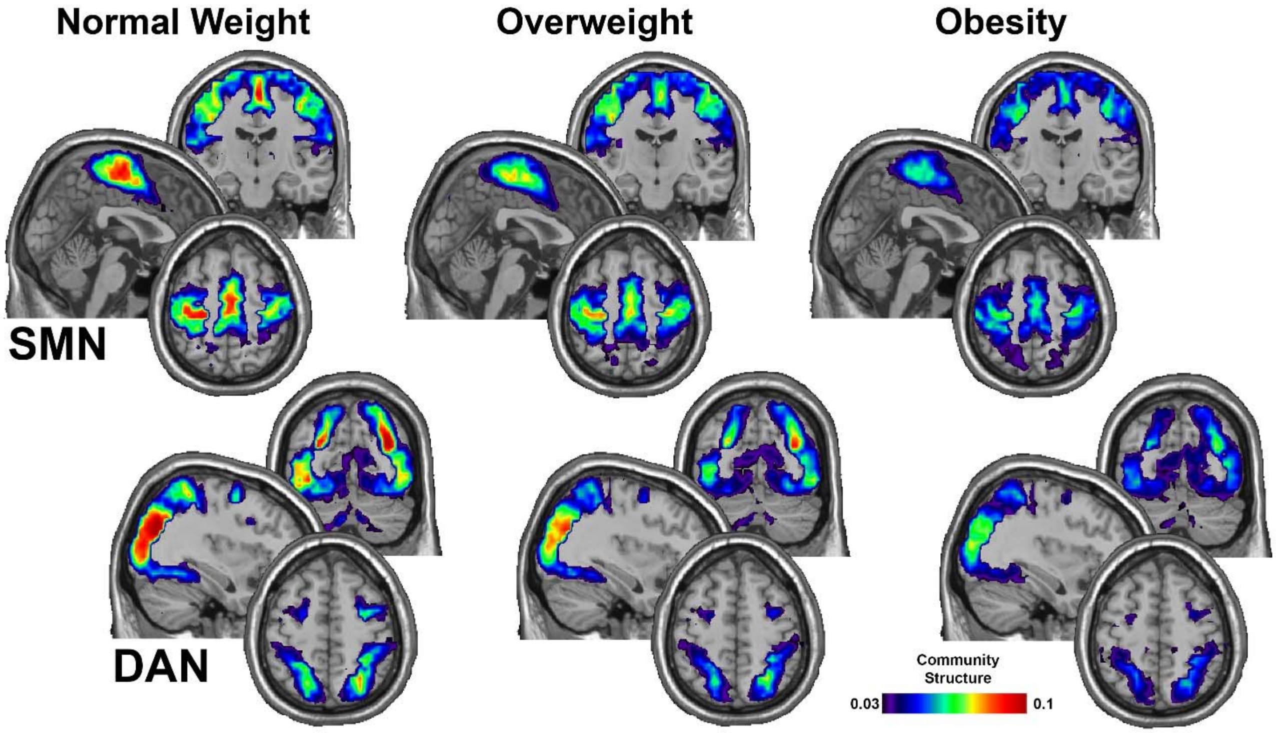

Figure S7: Community structure maps categorical groupings of BMI. Data were categorized as normal weight (18.5-24.9 kg/m^2), overweight (256-29.9 kg/m^2), and obesity ($\geq$ 30 kg/m^2). Images show the average community structure maps for each group. Note that an underweight category was not used as there were only two participants with a BMI < 18.5 kg/m^2. As found with the continuous regression models, the integrity of the community structure for both the SMN and DAN is highest for normal weight, followed by overweight, and lowest for obesity.

References

1. Ogawa S, Lee TM, Kay AR, Tank DW. Brain magnetic resonance imaging with contrast dependent on blood oxygenation. Proc Natl Acad Sci U S A. 1990;**87**:9868-9872.

2. Rejeski WJ, Rushing J, Guralnik JM, Ip EH, King AC, Manini TM*, et al.* The MAT-sf: identifying risk for major mobility disability. J Gerontol A Biol Sci Med Sci. 2015;**70**:641-646.

3. Rejeski WJ, Marsh AP, Anton S, Chen SH, Church T, Gill TM*, et al.* The MAT-sf: clinical relevance and validity. J Gerontol A Biol Sci Med Sci. 2013;**68**:1567-1574.

4. Neyland BR, Hugenschmidt CE, Lyday RG, Burdette JH, Baker LD, Rejeski WJ*, et al.* Effects of a Motor Imagery Task on Functional Brain Network Community Structure in Older Adults: Data from the Brain Networks and Mobility Function (B-NET) Study. Brain Sci. 2021;**11**.

5. Rorden C, Brett M. Stereotaxic display of brain lesions. Behav Neurol. 2000;**12**:191-200.

6. Avants BB, Tustison NJ, Song G, Cook PA, Klein A, Gee JC. A reproducible evaluation of ANTs similarity metric performance in brain image registration. Neuroimage. 2011;**54**:2033-2044.

7. Smith SM, Jenkinson M, Woolrich MW, Beckmann CF, Behrens TE, Johansen-Berg H*, et al.* Advances in functional and structural MR image analysis and implementation as FSL. Neuroimage. 2004;**23 Suppl 1**:S208-219.

8. Power JD, Barnes KA, Snyder AZ, Schlaggar BL, Petersen SE. Spurious but systematic correlations in functional connectivity MRI networks arise from subject motion. Neuroimage. 2012;**59**:2142-2154.

9. Hugenschmidt CE, Burdette JH, Morgan AR, Williamson JD, Kritchevsky SB, Laurienti PJ. Graph theory analysis of functional brain networks and mobility disability in older adults. J Gerontol A Biol Sci Med Sci. 2014;**69**:1399-1406.

10. Newman ME. Modularity and community structure in networks. Proc Natl Acad Sci U S A. 2006;**103**:8577-8582.

11. Delvenne JC, Yaliraki SN, Barahona M. Stability of graph communities across time scales. Proc Natl Acad Sci U S A. 2010;**107**:12755-12760.

12. Steen M, Hayasaka S, Joyce K, Laurienti P. Assessing the consistency of community structure in complex networks. Phys Rev E Stat Nonlin Soft Matter Phys. 2011;**84**:016111.

13. Mayhugh RE, Moussa MN, Simpson SL, Lyday RG, Burdette JH, Porrino LJ*, et al.* Moderate-Heavy Alcohol Consumption Lifestyle in Older Adults Is Associated with Altered Central Executive Network Community Structure during Cognitive Task. PLoS One. 2016;**11**:e0160214.

14. Moussa MN, Steen MR, Laurienti PJ, Hayasaka S. Consistency of network modules in resting-state FMRI connectome data. PLoS ONE. 2012;**7**:e44428.

15. Tomlinson CE, Laurienti PJ, Lyday RG, Simpson SL. A regression framework for brain network distance metrics. Netw Neurosci. 2022;**6**:49-68.

16. Benjamini Y, Hochberg Y. Controlling the False Discovery Rate - a Practical and Powerful Approach to Multiple Testing. J R Stat Soc B. 1995;**57**:289-300.

17. Benjamini Y, Hochberg Y. On the adaptive control of the false discovery fate in multiple testing with independent statistics. J Educ Behav Stat. 2000;**25**:60-83.
